# Lucio's phenomenon: exuberant case report and review of Brazilian cases*

**DOI:** 10.1590/abd1806-4841.20164370

**Published:** 2016

**Authors:** Rafael Henrique Rocha, Paulo Sergio Emerich, Lucia Martins Diniz, Marcela Bahia Barretto de Oliveira, Aline Neves Freitas Cabral, Ana Cristina Vervloet do Amaral

**Affiliations:** 1 Universidade Federal do Espírito Santo (UFES) - Vitória (ES), Brazil

**Keywords:** Lepromatous leprosy, combination chemotherapy, Therapy, Thrombosis

## Abstract

Lucio’s phenomenon is an uncommon reaction characterized by severe necrotizing cutaneous lesions that occurs in patients with Lucio’s leprosy and lepromatous leprosy. It is considered by some authors as a variant of type 2 or 3 reaction. Death can occur because of blood dyscrasia or sepsis. Precipitating factors include infections, drugs and pregnancy. We report a 31-year-old female patient exhibiting both clinical and histopathological features of lepromatous leprosy and Lucio’s phenomenon presenting favorable response to treatment. We complement our report with a literature review of the Brazilian cases of Lucio’s phenomenon published in Portuguese and English.

## INTRODUCTION

Leprosy is a chronic infectious disease caused by *Mycobacterium leprae* and transmitted by inhalation of bacilli after close and frequent contacts with untreated patients.^1^

Lucio’s phenomenon (LP) is an uncommon reaction characterized by severe necrotizing cutaneous lesions that occur in patients with Lucio’s leprosy and lepromatous leprosy (LL).^2^ It is considered by some authors as a variant of type 2 or 3 reaction. Although Brazil has the world’s second highest incidence of leprosy (33,303 cases), few cases of LP are reported in the Brazilian literature.^3^

We report a case of extensive LP in a patient with LL and provide a table containing data of Brazilian cases of LP published in the literature in both Portuguese and English.

## CASE REPORT

We report a 31-year-old female patient, from São Mateus (ES) that presented to the emergency room of University Hospital Cassiano Antonio Moraes (HUCAM) in Vitória (ES) with extensive severe ulcerative-necrotic lesions involving the abdomen, upper and lower limbs. Lesions first appeared on the legs one year before with vasculitis diagnosed at another hospital. Treatment consisted of prednisone 80mg/day, progressing to remission. The daily use of the medication continued for four more months, and after she dropped out the treatment.

**Figure 1:**
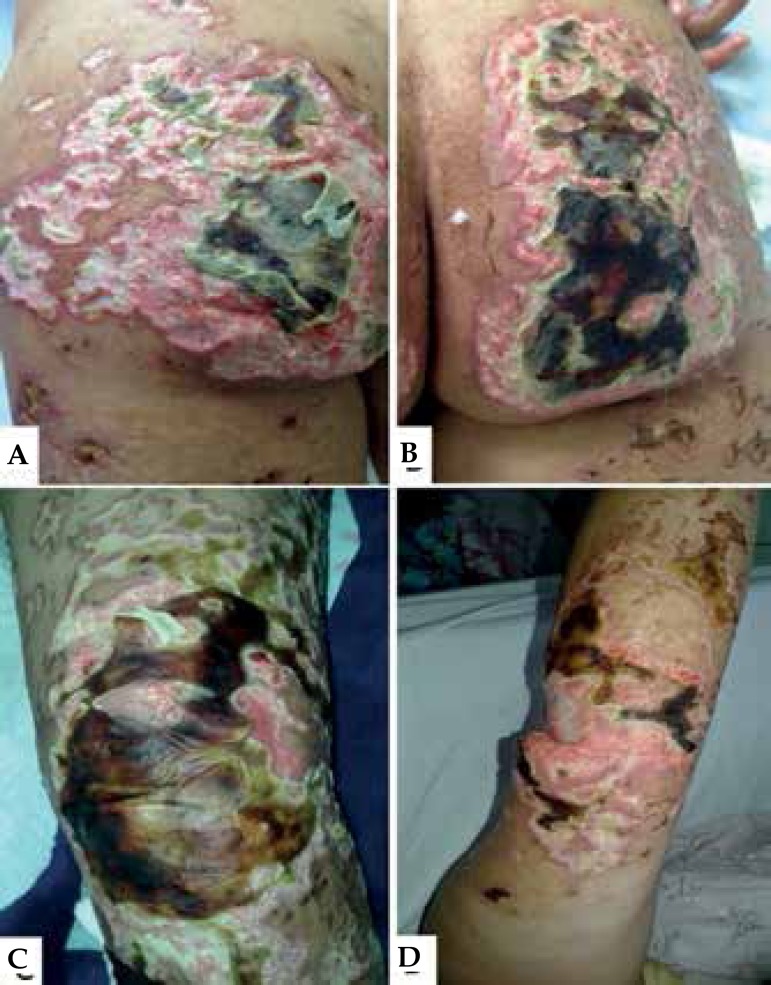
**A** and **B)** bilateral necrosis on the left and right buttocks, respectively. **C)** necrosis on the right knee. **D)** Necrosis on the right elbow

**Figure 2:**
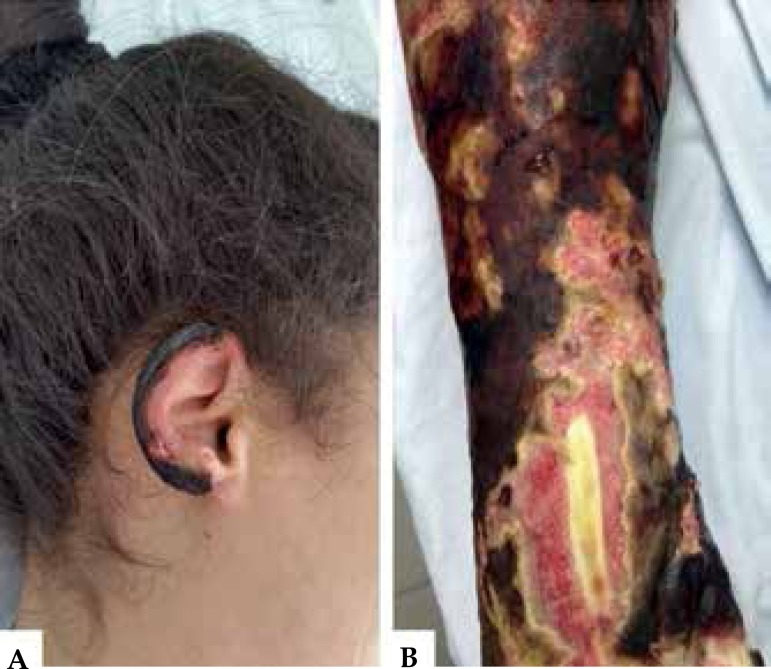
**A)** Necrosis of the right ear **B)** Traduzir para: **B)** Necrosis and tendon exposure on the left leg

The patient reported a 2-month history of worsening of skin lesions, glycemic decompensation and signs of secondary infection. She was referred to the emergency room of HUCAM for investigation. On admission, the patient was in a bad general state, pale, and febrile (38.2° C). Dermatological examination revealed extensive aspect of shallow polygonal ulcers with fibrinonecrotic base and irregular erythematous edges, mostly present on the limbs and less frequently on the abdomen and face. Bilateral necrosis on the ear, elbows, buttocks and toes was identified. The left ankle revealed tendon exposure (Figures 1 and 2).

Laboratory tests showed anemia (hemoglobin: 8.6 g/dL; hematocrit: 27.2%; MCV: 71.4fL); normal white blood cell count (9760/mm³; bands: 5%); high levels of C-reactive protein (206mg/L); negative serology for viruses B, C and HIV; VDRL 1:4; negative FAN, anti-ENA and anti-DNA; Normal C3 and C4; positive results for IgM anticardiolipin (234,8GPL-U/ml); and negative IgG.

**Figure 3:**
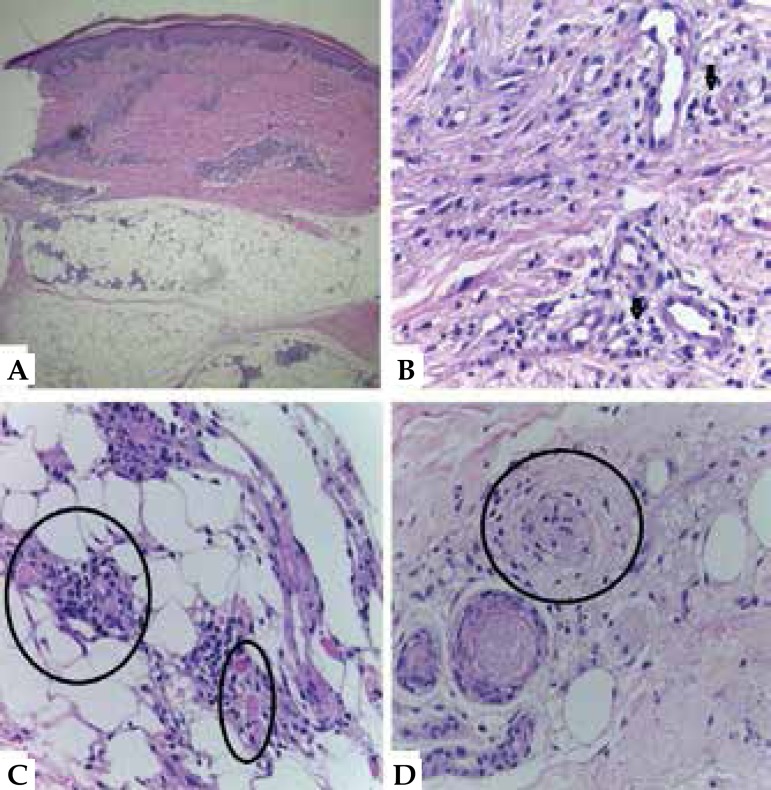
**A)** Inflammatory infiltrate affecting the dermis and hypodermis (Hematoxylin - eosin, x40). **B)** Perivascular lymphohistiocytic infiltrate with multiple Virchow’s cells (see arrows)(Hematoxylin - eosin, x100). **C)** Thrombosis of vessels in the hypodermis (see circles)(Hematoxylin - eosin, x100). **D)** Endothelial proliferation with occlusion of the vessel lumen (see circle), (Hematoxylin - eosin, x100)

**Figure 4:**
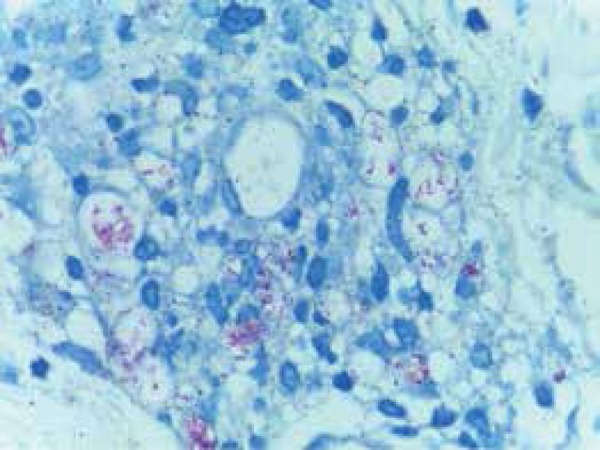
Numerous bacilli on the lumen and vessel wall invading endothelial cells. BAAR 6/6+ globi 3/3+. Ziehl Neelsen coloring (Hematoxylineosin, x400)

After considering the hypothesis of LP, we performed a biopsy of the edge of an ulcer on the right thigh. Histopathology revealed superficial and deep inflammatory perivascular lymphohistiocytic infiltrate, with Virchow’s cells and vascular thrombosis, bacilli on the lumen, on the vessel wall and invading endothelial cells, BAAR 6/6+, globi 3/3+, confirming the diagnosis of LL with LP (Figures 3 and 4).

**Figure 5:**
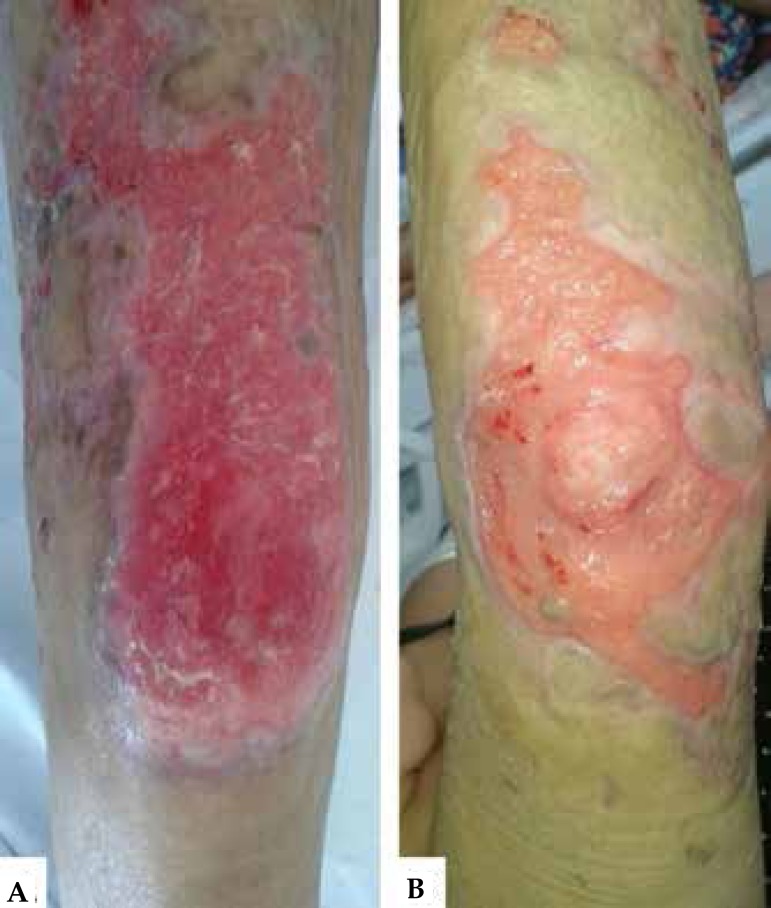
Improvement of lesions on the **A)** right knee and **B)** right elbow - 45 days after the start of the treatment

The patient received clinical care, was treated with antibiotics (meropenem and vancomycin), underwent chemical and surgical debridement of the lesions, and was submitted to multibacillary multidrug therapy (MDT-MB) associated with pentoxifylline. We observed significant improvement of skin lesions 45 days after the start of treatment (Figure 5).

## DISCUSSION

Lucio’s phenomenon (LP) is an uncommon reaction characterized by severe necrotizing cutaneous lesions that occurs in patients with Lucio’s leprosy and lepromatous leprosy. The syndrome is almost exclusively limited to Mexican and Central American patients, although it is rarely reported in Cuba, South America, the United States, India, Polynesia, South Africa and Southeast Asia.^2^ It is characterized by painful, slightly infiltrated, erythematous macules outbreaks and hemorrhagic blisters that develop with central necrosis and ulceration.^4^

Necrotic, jagged-edged, geometric-shaped ulcers are classic features. Lesions mainly affect extremities, leaving atrophic star-like scars. Face and trunk involvement is rarely found. The syndrome usually occurs in untreated leprosy patients or in patients taking irregular treatment. It manifests itself 1-3 years after the onset of the disease. Fever may be due to secondary infection – commonly present in cases of LP – or occasionally due to a systemic manifestation of the disease (as well as anemia).^4^

LP was considered the cause of secondary antiphospholipid antibody syndrome in cases reporting positive results for IgM anticardiolipin antibodies and negative IgG.^5^

Severity of LP is related to delayed initiation of therapy progressing to death due to the blood dyscrasia or sepsis. Precipitating factors include infections, drugs and pregnancy.^6^

The most accepted hypotheses for the pathogenesis of LP refers to the severe congestive vascular reaction, usually hemorrhagic. Bacterial liposaccharides would stimulate active macrophages to release IL-1 and TNF-α. Those products would act on endothelial cells producing prostaglandins, IL-6 and coagulation factor-III, thus causing the formation of thrombi inside the vessels and also promoting tissue necrosis.^7,8^

Histopathology remains poorly defined and there is no agreement among the authors on the presence or absence of leukocytoclastic vasculitis and on the terminology used. The most commonly described features are: the presence of acid-fast bacilli in the dermis; perivascular, with wall invasion of vessels and endothelial cells; decrease in the vascular lumen by endothelial proliferation and thrombosis of vessels in the dermis and hypodermis; inflammatory infiltrate with lymphocytes and neutrophils; and perivascular histiocytic granulomas.^8^

The literature adopts three criteria to define LP: skin ulceration, vascular thrombosis and blood vessel wall invasion by bacilli.^6,8^

Due to the small number of LP cases described in the literature, there is no consensus on the best treatment option. Currently, the most commonly adopted treatments are multibacillary multi-drug therapy for leprosy and antibiotics for secondary infections. Controversy still exists over the use of corticosteroids.^6,8^ Some studies report the use of pentoxifylline associated with leprosy treatment due to its antiplatelet effect and blood flow improvement.^9^

As shown in chart 1, despite the scarcity of published cases, it is clear that LP represents a severe reaction with significant mortality rates, mostly associated with pregnancy and secondary infection.^2,4,8,9,10^ MDT-MB was the most common treatment, but we identified differences in the association or not of other medicines with the treatment. There is no consensus on the histopathology of LP. We observed a higher incidence of the disease in São Paulo state. Clinical and histopathological features of the present case were consistent with LP and our patient showed a good response to treatment. We report the first case of LP published in Espirito Santo state.

**Chart 1:** Cases of Lucio’s phenomenon published in Brazil

References	Case	Sex	Age	Race	State of origin	Pregnancy - puerperium	Histopathology	Treatment	Death
Souza *et al*^9^	1	H	45	-	SP	-	BAD/NEP	PQTA/CT	S
							TRV/IVB	ATB/TL	
Souza *et al*^9^	2	H	51	-	SP	-	BAD/NEP	PQT/CT	N
Souza* et al*^9^	*3*	*H*	*65*	*-*	*SP*	*-*	*BAD/NEP*	*PQT/CT*	*N*
							TRV/IVB	ATB/PTX	
Souza *et al*^9^	4	M	45	-	SP	N	BAD/NEP	PQT/PTX	N
							TRV/IVB		
Buffon *et al*^10^	5	M	25	B	SP	S	BAD/NEP	PQT/CT	S
							IVB	ATB	
Helmer *et al*^11^	6	M	27	B	PR	S	BAD/VAL	PQT/CT	N
Costa *et al*^4^	7	M	34	B	DF	N	BAD/NEP	PQT/CT	S
							TRV/IVB	ATB	
Bernard *et al*^12^	8	M	28	N	SP	S	BAD/NEP	PQT/CT	N
							TRV/IVB		
Campos *et al*^13^	9	M	67	B	SP	N	BAD/TRV	#Diagnóstico	S
							IVB/VAL	por autópsia	
Monteiro *et al*^8^	10	H	61	P	SP	-	BAD/NEP	PQT/CT	N
							TRV/IVB	ATB/TL	
Peixoto *et al*^2^	11	H	63	P	RJ	-	BAD/VAL	PQT	

H-Man; M-Woman; B-White; P-Mulatto; N-Black; PQTA-alternative MDT-MB (clofazimine and ofloxacin); PQT/MB-multibacillary multidrug therapy; CT-corticosteroid therapy; PTX-pentoxifylline; ATB-antibiotic therapy; TL-thalidomide; VAL leukocytoclastic vasculitis; TRV-thrombosis of vessels, BADBAAR in the dermis; IVB- blood vessel wall and endothelial cells invasion by bacilli; NEP-ischemic necrosis of the epidermis; S-Yes; N-No
